# Research Misconduct: A Report from a Developing Country

**Published:** 2017-10

**Authors:** Majid KHADEM-REZAIYAN, Maliheh DADGARMOGHADDAM

**Affiliations:** Dept. of Community Medicine and Public Health, Mashhad University of Medical Sciences, Mashhad, Iran

**Keywords:** Research misconduct, Fabrication, Falsification, Plagiarism

## Abstract

**Background:**

Cheating rate is rising and engages newer methods. This study performed to estimate the rate of research misconduct in the thesis of undergraduate and postgraduate medical students in 2015.

**Methods:**

In this cross sectional study, all undergraduate and postgraduate medical students graduated during the study period in 2015, from the School of Medicine, Mashhad University of Medical Sciences, Mashhad, Iran were asked to fill a small checklist anonymously. It consisted of two demographic questions and two other ones for estimation of research misconduct. All three major types of research misconduct were explained in the checklist. We used the Randomized Response Technique for sensitive question in this survey. We asked the respondent to choose one question randomly and answer to it. The probability of selection of each question was equal.

**Results:**

There were 149 filled questionnaires out of which 44 (31%) were graduated for General Practitioner, 63 (44%) for Residency, 31(21%) for Master Degree and 6 (4%) for Ph.D. Fifty-two percent (75) were male. More than half of participants were graduated between 2011 and 2012. The majority of participants were native (104, 81%). Undergraduate students had an estimation of 19% research misconduct in performing the thesis while this was 26% of postgraduate students. Males were nearly two times comparing to females in this issue (30% vs. 16%).

**Conclusion:**

This high estimation must be considered in future policy making about observing strictly on researches.

## Introduction

Cheating is a worldwide phenomenon that involves educational systems (1–3). Cheating rate is rising and engages newer methods. Medical science is not excluded from this issue and reports of research misconduct by medical practitioners go back for at least a century (4). Scientific fraud can distract the search for truth, and it contaminates the record of scientific literature (5). Research misconduct has a great burden both in economic and human terms (6). In 2000, the US Office of Research Integrity provided a concise definition of research misconduct as “fabrication, falsification or plagiarism in proposing, performing or reviewing research or in reporting research” (7). The Medical Research Council of United Kingdom defines misconduct as “fabrication, falsification, plagiarism, or deception in proposing, carrying out or reporting results of research and deliberate, dangerous, or negligent deviations from accepted practice in carrying out research” (8).

Fabrication is defined as the invention of data or information, falsification is defined as the alteration of the observed result of a scientific experiment and plagiarism is defined as taking someone else’s work without attributing the source and claiming it to be one’s own (9).

Although there is a convincing amount of research on scientific fraud around the world, few reports have come from developing ones like Iran (10). In one of these unpublished research projects, 50% of students declared that they had committed fabrication and falsification in their thesis (11). In another one, some real cases of misconduct happened in one of the main medical universities of Iran have been discussed (12). Fabrication and falsification were estimated to be 37% and 40% of thesis, respectively. Plagiarism was also estimated to be 25%–50% (13).

A report has shown that Iran (6.60), India (5.68), Turkey (5.38), South Korea (3.59), and China (2.00) had higher ratios of “publication misconduct” to “distrust data or interpretations” than other countries. Because plagiarism is a major component of the publication misconduct category, the English language barrier may be at least partially responsible for the high ratios of Iran, Turkey, South Korea and China (6).

One of the main causes of this underreporting is that researchers and their institutions often react with denial, and may be anger when faced with an accusation of research misconduct (4).

The promulgation of codes of ethical research practice and the provision of training in research ethics and especially promoting innate moral values of the individuals can reduce this ugly behavior (4). However, for proper education, basic information is needed. Therefore, this study was performed to cover this gap about proper estimation of research misconduct in thesis of undergraduate and postgraduate medical students.

## Methods

This cross sectional study was performed in 2015. All undergraduate and postgraduate medical students from the School of Medicine, Mashhad University of Medical Sciences, Mashhad, Iran graduated during the study period were asked to fill a small checklist anonymously. It consisted of two demographic questions and two other ones for estimation of research misconduct. All three major types of research misconduct were explained in the checklist.

We used the Randomized Response Technique for sensitive question in this survey. Warner proposed the randomized response method as a technique that reduces potential bias of nonresponse and social desirability when asking questions about sensitive behaviors and beliefs (14). The method asks respondents to use a randomizing selection whose outcome is not obvious for the researcher. In this method, the respondents may be more inclined to answer truthfully. Two assumptions should be met in this method: (A) the randomization distribution is known to researchers, and (B) respondents comply with the instructions and answer the sensitive question truthfully when prompted. In the unrelated question design, randomization determines whether a respondent should answer a sensitive question or an unrelated, non-sensitive question (15). Unlike the other designs of random response, the unrelated question design introduces an unrelated question to increase respondents’ compliance with survey instruction (16).

We asked the respondent to chose one question randomly and answer to it. The probability of selection of each question was equal. The sensitive question was: “Have you committed a research misconduct (Fabrication, falsification or plagiarism) for your thesis?” The unrelated question was “Did you born in summer?” and there was a determined probability for this question. This checklist was filled in the final step before completing the official process of graduation. This research was approved by Deputy Research of Mashhad University of medical sciences (931603).

## Results

Totally there were 149 filled questionnaires out of which 44 (31%) were graduated for General Practitioner, 63 (44%) for Residency, 31(21%) for Master Degree and 6 (4%) for Ph.D. Fifty-two percent (75) were male. More than half of participants were graduated from 2011–2012. The majority of participants were native (104, 81%).

**Fig. 1:**
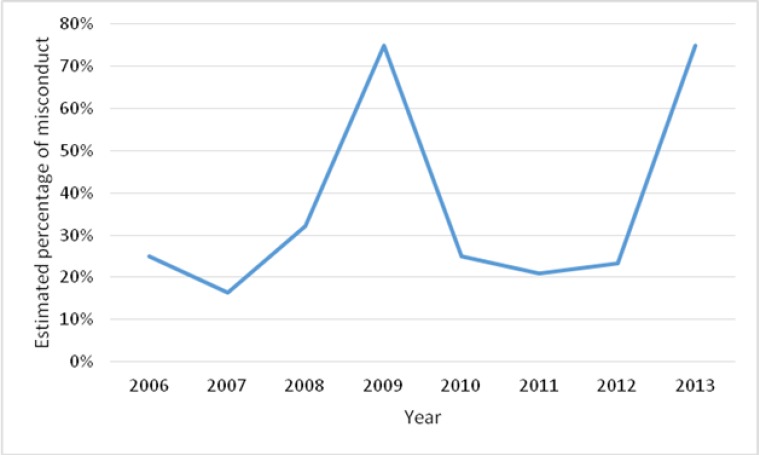
Estimated percentage of research misconduct in performing thesis based on the acceptance year in the medical university

Undergraduate students had an estimation of 19% research misconduct in performing the thesis while this was 26% of postgraduate students. Males were nearly two times comparing to females in this issue (30% vs. 16%). The trend of research misconduct has a peak is shown in Fig. 1.

Research misconduct was nearly twice in students accepted as guest in this university comparing to native ones (50% vs 26%). However, the least estimated misconduct belonged to transferred students (13%).

## Discussion

According to the main results of this study, undergraduate students had an estimation of 19% research misconduct in performing the thesis while this was 26% of postgraduate students. Males were nearly two times comparing to females in this issue (30% vs. 16%).

In a systematic review and meta-analysis study that was published in 2009, a pooled weighted average of 1.97% (N=7, 95%CI: 0.86–4.45) of scientists accepted to have fabricated, falsified or modified data or results at least once. In surveys about the behavior of colleagues, admission rates were 14.12% (N = 12, 95% CI: 9.91–19.72) for falsification and up to 72% for other questionable research practices (17). Asking about others behaviors is an indirect method and is recommended for asking sensitive questions. This estimation was much closer to our estimation.

The true estimation of fraud in medical research is difficult for many reasons. Direct estimation via a survey of investigators, those who commit fraud are not likely to be forthcoming about having done. The underreport lead to iceberg phenomenon (18).

“When conducting medical research, one must abide by the ethical and moral obligations as outlined by the Nuremberg code in 1947 (19) and the subsequent Declaration of Helsinki 1964 (and later revised in 2002) (20, 21), which explain the responsibilities of scientists and physicians when conducting medical research on humans.” However, despite these guides, there is a long history of fraud in medical researches (22–24).

A majority of respondents (biostatisticians) was reported knowing of at least one serious breach of fraudulent projects in the past 10 years (25).

A total of 3247 mid-career were surveyed (majority at the associate professor level or above) and early-career scientists (majority at post-doctoral level) working in the United States and showed that the percentage engaging in such activity such as falsification or fabricating data, was low (<2%) (26) that it may result in the knowledge of ethical research codes. However, surprisingly, in our study, undergraduate students had an estimation of 19% research misconduct in performing the thesis while this was 26% of postgraduate students. It may due to more statistical knowledge in postgraduate student that enable them to do data making the other reason maybe the restricted time and overloaded work in residency program.

Few reasons were suggested for this phenomenon, but the most important underlying factors are to be successful in science and also a fear of failure (27).

Academic pressure, personal desire for fame, “sloppy” science, financial gain, and inability to determine right from wrong are a number of reasons why research misconduct takes place. Ethical standards need to be made clear and there needs to be an alleviation of pressure on researchers, as well as greater control on medical researchers.

“It is time to consider what aspects of the research environment are most salient to research integrity, which aspects are most amenable to change, and what changes are likely to be most fruitful in ensuring integrity in science” (26).

## Conclusion

Undergraduate students had an estimation of 19% research misconduct in performing the thesis while this was 26% of postgraduate students. This high estimation must be considered in future policy making about observing strictly on researches.

## Ethical considerations

Ethical issues (Including plagiarism, informed consent, misconduct, data fabrication and/or falsification, double publication and/or submission, redundancy, etc.) have been completely observed by the authors.
